# Repetitive application of remote ischemic conditioning (RIC) in patients with peripheral arterial occlusive disease (PAOD) as a non-invasive treatment option: study protocol for a randomised controlled clinical trial

**DOI:** 10.1186/s12872-022-02795-3

**Published:** 2022-08-04

**Authors:** Lars Hummitzsch, Luisa Voelckers, Melanie Rusch, Jochen Cremer, Martin Albrecht, René Rusch, Rouven Berndt

**Affiliations:** 1 Department of Anesthesiology and Intensive Care Medicine, University Hospital of Schleswig-Holstein, Kiel, Germany; 2 Clinic of Cardiovascular Surgery, University Hospital of Schleswig-Holstein, Campus Kiel, Arnold-Heller-Str. 3, Hs C, 24105 Kiel, Germany; 3 Vascular Research Center, University Hospital of Schleswig-Holstein, Kiel, Germany

**Keywords:** Remote ischemic conditioning (RIC), Repeated RIC, Chronic RIC, Peripheral arterial occlusive disease (PAOD), Randomized controlled trial

## Abstract

**Background:**

The best medical treatment (BMT) for most patients with early stage of peripheral arterial occlusive disease (PAOD) is often limited to gait training and pharmacological therapy besides endovascular surgery. The application of remote ischemic conditioning (RIC) has been described as a promising experimental strategy for the improvement of therapeutic outcome in cardiovascular disease but has not proven beneficial effects in clinical practice and treatment of PAOD yet.

**Methods:**

Here we describe a prospective, randomized trial for the evaluation of possible effects of repeated application of RIC in patients with PAOD. This monocentric study will enrol 200 participants distributed to an intervention group receiving RIC + BMT and a control group only receiving BMT for four weeks. Patients are at least 18 years of age and have diagnosed PAOD Fontaine stage II b. Pain-free and total walking distance will be measured via treadmill test (primary endpoints). In addition, ankle-brachial index (ABI) and quality of life (QoL) will be assessed using the SF-36 and VascuQoL-6 questionnaire. Moreover, evaluation of markers for atherosclerosis, angiogenic profiling and mononuclear cell characterization will be performed using biochemical assays, proteome profiling arrays and flow cytometry (secondary endpoints).

**Discussion:**

Our prospective, randomized monocentric trial is the first of its kind to analyse the effects of chronic and repetitive treatment with RIC in patients with PAOD and might provide important novel information on the molecular mechanisms associated with RIC in PAOD patients.

*Trial registration:* Prospectively registered in the German Clinical Trials Register (Deutsche Register Klinischer Studien) Registration number: DRKS00025735; Date of registration: 01.07.2021.

## Background

Due to demographic and lifestyle changes in modern society Peripheral Arterial Occlusive Disease (PAOD) has become one of the most common diseases worldwide [1]. For most patients suffering from PAOD with moderate to severe symptoms only pharmacological therapy and gait training remain as a therapeutic option until the course of the disease leads to unavoidable endovascular and open vascular surgical procedures. Accordingly, multiple advanced therapeutic approaches have been invastigated in order to overcome the actual limitations in the treatment of PAOD.

The concept of remote ischemic conditioning (RIC) has been evaluated as a promising strategy for patients with cardiovascular disease and may offer new treatment and rehabilitation options for patients with PAOD. RIC as a therapeutic strategy includes repeated, non-lethal episodes of ischemia to an organ or limb exerting protection and potentially faster regeneration from ischemia–reperfusion (I/R) injury [2]. This effective, simple and low-risk procedure can be easily induced by transient occlusion of blood flow with a blood pressure cuff located on the upper arm [3, 4].

However, only small and heterogenous studies have evaluated the potential effects of RIC on patients with PAOD. Unfortunately, most of these studies employed a single and limited application of RIC [5, 6]. Recent studies suggested that daily repeated RIC (chronic RIC; cRIC) might be more effective for cardiovascular protection than a single RIPC application (Chong et al. 2019). Therefore, the here described study aimed to investigate for the first time the long-term application of RIC on a homogeneous cohort of patients with PAOD Fontaine stage II b and investigate underlying molecular mechanisms.

## Methods

### Hypothesis

We hypothesize that long-term, repetitive application of RIC in combination with best medical treatment (BMT) including systematic gait training and pharmacological standard therapy will lead to longer painless and total walking distance, better quality of life (QoL) and an enhanced ankle-brachial index (ABI), when compared to standard BMT therapy in the control group.

### Study registration and ethical approval

The here described study protocol has been approved by the institutional ethic committee of the University Hospital Schleswig–Holstein (UKSH) (protocol number: D563/20) and has been registered in the *German Clinical Trials Register* (Deutsche Register Klinischer Studien = DRKS; Registration number: DRKS00025735). The study protocol follows the Standard Protocol Items: Recommendations for Interventional Trials (SPIRIT) guidelines. All patients will be assigned randomly 1:1 into two different groups through random permuted blocks. Enrolment of patients will be supported by two study nurses, guided by the cardiovascular outpatient department of the University Hospital Schleswig–Holstein and documented by a study diary. Written declaration of consent will be available for the enrolled patients.

### Study design

**Fig. 1:**
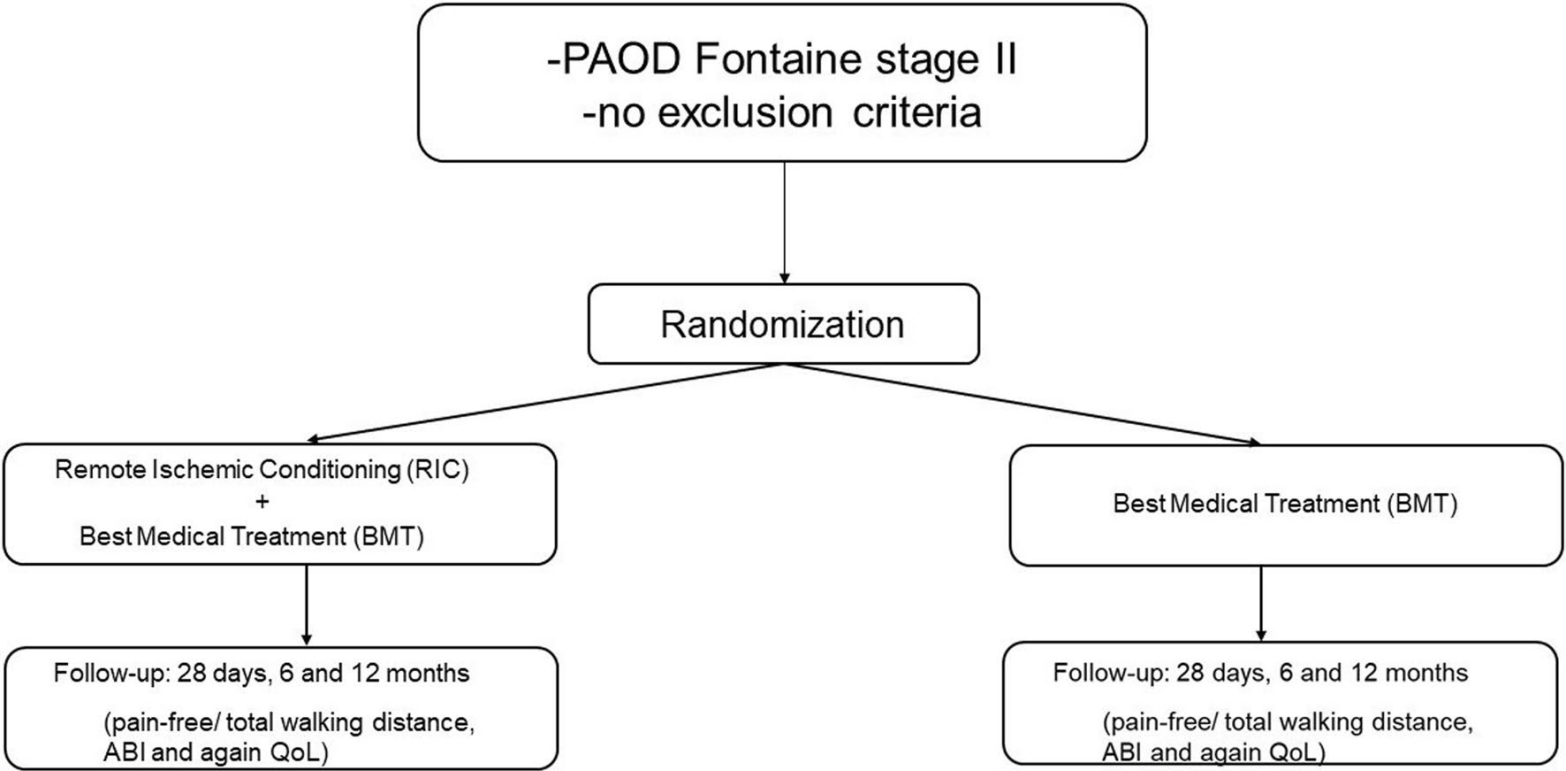
SPIRIT Flow diagram for inclusion and treatment. ABI = ankle-brachial index; BMT = best medical treatment; PAOD = peripheral arterial occlusive disease; remote ischemic conditioning (RIC), QoL = quality of life

**Fig. 2:**
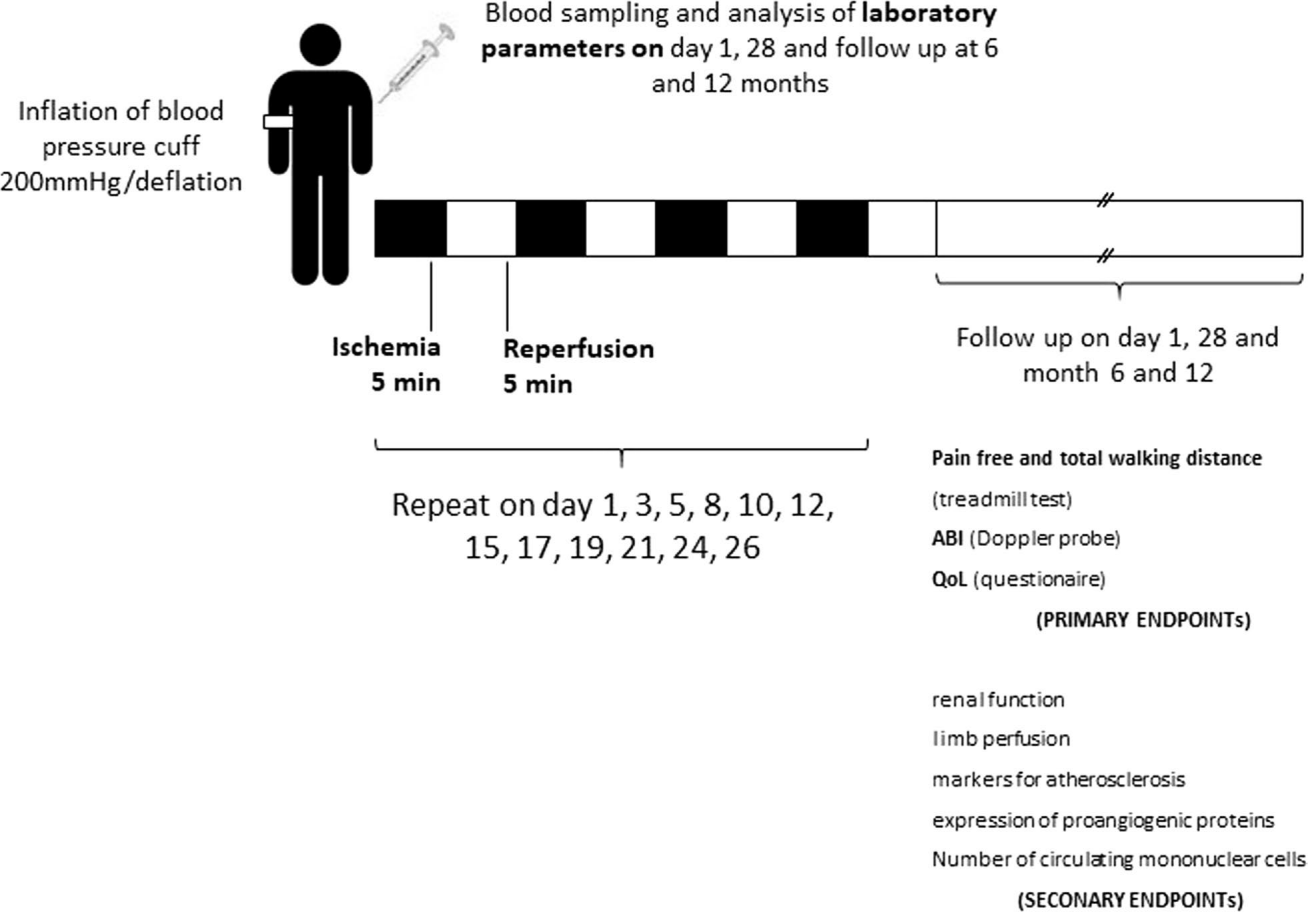
Schematic depiction of the study and long-term follow-up

This study is a randomized, controlled monocentric trial, as presented in Fig. 1. For a schematic representation of the study, please refer to Fig. 2.

### Patients

A total of 200 patients (minimal number to treat 120) with diagnosed PAOD Fontaine stage II b and aged over 18 years will be randomised if written consent is given.

### Primary endpoints

Primary endpoint of the study is the improvement of the pain-free and total walking distance after RIC + BMT compared to BMT in the control group, respectively. The determination of the pain-free and total walking distance and classification according to the Fontaine classification stage I-IV will be assessed by a standardized treadmill-test at 3.2 km/h (2 miles/h) and an incline of 12% on the treadmill. Moreover, improvement of the ankle-brachial index (ABI) and the Quality of Life (QoL) will be determined by the SF-36 and VascuQoL-6-test (Fig. 1).

### Secondary endpoints

Secondary endpoints will be assessed to determine the correlation between the development of collateral blood vessels and clinical symptoms and to identify any other beneficial effects of RIC in the PAOD clinical scenario. The following secondary objectives will be assessed:

- Influence of RIC on renal function, serum creatinine (improvement of pre-existing renal insufficiency)
- Determination of the effects of RIC on limb perfusion using thermal imaging camera
- Comparison of laboratory chemical markers for atherosclerosis HS-CRP, cholesterol, IL-6, Il-1, fibrinogen, homocysteine, lipoprotein-(a)
- Molecular biology and protein biochemistry analyses on the influence of RIC on the protein expression profile of proangiogenic and ischemia-protective factors
- Number of circulating mononuclear cells (CD14, CD26, CCR2, CX3CR1, CD105, TIE2)

### Eligibility criteria

Patients meeting the following criteria will be *included* in the study protocol:

1. Occurrence of PAOD II b for at least four weeks
2. No surgical or interventional treatment within the last to weeks
3. Written informed consent

While patients with the following conditions will be *excluded* from the study protocol:

1. Age < 18 years > 75 years
2. Current participation in another clinical intervention study
3. Participation in another clinical intervention study within the last 30 days
4. Cognitive/language barriers, comprehension problems during the informed consent process
5. Severe renal and hepatic insufficiency
6. Severe COPD (FeV1 ≤ 50%)
7. Existing drug and alcohol abuse
8. Neurological diseases (Parkinson's disease, multiple sclerosis, epilepsy, Alzheimer's disease)
9. Acute infections
10. Pronounced PAOD of the upper extremities
11. Polyneuropathy of the upper extremities
12. Arterio-venous fistula (shunt) or lymphedema of the upper extremities
13. Myocardial infarction < 2 months
14. Stroke < 2 months
15. Malignancy
16. Pregnancy
17. Lack of compliance, > 3 missed RIC applications

### Randomization

Central randomization will be performed by block randomization with use of an automated web-based randomization tool.

### Study intervention

Repetitive, non-injurious ischemia will be induced by inflation and deflation of a blood pressure cuff at the upper limbs with a defined pressure of 200 mmHg. The mechanism of tissue/organ protection by RIC is mainly based on activation of neuronal efferences, systemic anti-inflammatory responses and the release of humoral factors via the bloodstream [3, 4]. Additionally, RIC might have a beneficial effect on chronic remodelling processes and angiogenesis which is highly relevant for treatment of PAOD [2, 3, 5, 6].

Application of RIC has been classified as a non-painful intervention, but with slightly unpleasant symptoms (numbness, tingling paraesthesia, which are completely reversible after a short time). Conceivable side effects of RIC such as thrombosis, arterial plaque rupture, nerve and vascular injury did not occur in any patient in the large phase III studies conducting a RIC protocol [8, 9]. Here we describe a repetitive, chronic RIC application for 3 × 5 cycles (each with 5 min ischemia and 5 min reperfusion) on day 1, 3, 5, 8, 10, 12, 15, 17, 19, 21, 24, 26. The treatment and follow-up will be supported by two study nurses and a study diary to increase the patient compliance.

### Adverse events and stop criteria

Adverse events (AE) are defined as any undesirable event occurring to the study participants during the clinical trial, whether or not considered related to the investigational treatment. All AEs and serious AEs reported spontaneously by the subject or observed by the investigator or his staff will be recorded using an AE form during admission and throughout 24 months of follow-up.

If stop criteria occur, study participants will immediately stop RIC treatment and the cardiovascular surgery outpatient department clinic or study investigators will be informed. The Ethic Committee will be notified immediately of any incident.

The following stop criteria are designated for RIC:

- persistent swelling and redness of the arm
- persistent paraesthesia, despite termination of RIC
- persistent pain, despite termination of RIC
- formation of large hematomas in the skin after RIC

### Statistical analysis

All calculations for the planned number of cases were performed using the G*power 3.1 software (http://www.gpower.hhu.de) and StatMate (Graphpad, San Diego, USA). Data will be analysed with the latest version of the Graphpad prism software (Graphpad Software, San Diego, USA). A drop-out rate of 20% was calculated for the trial design and a minimum of 120 patients should be included. D'Agostino‐Pearson or Kolmogorov‐Smirnov test will be used for normality testing. Paired respectively unpaired student's t test will be used for metric and Wilcoxon-Mann–Whitney-Test will be used for non-metric data with regard to the primary and secondary endpoints.

### Follow-up

The trial will be supported by two study nurses respectively research assistants during the study course. Any complications will be recorded and additionally the patients will fulfil a mandatory study diary to increase compliance. At day 28 all patients will undergo a second treadmill test, ABI and final interviews for assessment of QoL (Fig. 2). Blood samples will be collected for further analysis of secondary endpoints. A further six months and twelve months follow-up will be provided by investigation of pain-free walking distance, ABI and again QoL to observe long-term effects of RIC in patients with PAOD.

### Trial status

After approval of the ethical committee of the University Hospital Schleswig–Holstein a first pilot study has been conducted prior to the main study. Currently 20 patients have been enrolled and the completion of the main trial is scheduled for December 2023.

## Discussion

Optimized treatment of PAOD remains a major and interdisciplinary challenge in current medicine [10]. Recent clinical studies have provided evidence that RIC could be a beneficial treatment option for cardiovascular disease in general but have also shown that large heterogenous trials and patient cohorts might not be the tools of first choice [5, 6, 8, 9]. Therefore, we here describe a trial design that will clarify the potential influence of RIC on relevant outcome parameters and QoL in patients with PAOD stage II Fontaine. Until today, only a few clinical studies have investigated the potential influence of RIC on PAOD with a limited number of patients and single respectively limited treatments [5, 6]. In contrast we hypothesised that chronic, repeated application of RIC might be necessary to generate PAOD-related therapeutic effects such as angiogenesis and collateral growth based on the knowledge that cardiovascular remodelling is often based on repeated stimuli [11, 12]. We have therefore conducted a study design that includes 3 × 5 RIC cycles per treatment day over a time period of 28 days. To the best of our knowledge this will be the first study to investigate this kind of repetitive RIC treatment in patients with PAOD.

A particular strength of this study might be the close monitoring of the patients during the trial which is represented by the employment of study nurses/research assistants providing weekly telephone/video feedbacks and a study diary. These aspects could be key factors to increase compliance and avoid the relatively high dropout rates described in similar trials [13].

Further, the here described study design not merely includes the clinical evaluation of the study patients but also conducts an experimental set-up taking into account the analysis of pro-angiogenetic protein levels and cellular transformation of mononuclear cell lines potentially influenced by RIC treatments [14, 15]. Accordingly, this study will provide insights not only in clinical outcome but also in molecular mechanisms associated with the effects of RIC treatment [16]. This monocentric trial design might represent a blueprint to enable further potential multicentric studies in patients with PAOD undergoing repetitive RIC application.

## Data Availability

All relevant data is included in the manuscript.

## Abbreviations

ABI: Ankle-brachial index
BMT: Best medical treatment
FC: Flow cytometry
PAOD: Peripheral arterial occlusive disease
RIC: Remote ischemic conditioning
QoL: Quality of life
